# Development of sustainable pediatric heart surgery program with international assistance model in two centers: 5 years results

**DOI:** 10.1186/1749-8090-8-S1-O265

**Published:** 2013-09-11

**Authors:** I Polivenok, A Nokhrin, F Molloy, C Gilbert, O Buchneva, M Danton, A Dodge-Khatami, K Drozdovski, V Bojko, W Novick

**Affiliations:** 1 Institute of General and Urgent Surgery, Kharkov, Ukraine; 2 Kuzbass Cardiology Center, Kemerovo, Russian Federation; 3 International Children’s Heart Foundation, Memphis, TN, USA; 4 Department of Surgery, University of Tennessee Health Sciences Center, Memphis, TN, USA

## Background

Surgery for Congenital heart disease has been slow to develop in parts of the former Soviet Union. We describe the impact of our 5-year surgical collaborative assistance program between the International Children’s Heart Foundation (ICHF) and two different developing centers in Russia and Ukraine.

## Methods

Data were analysed from ICHF, Kharkov and Kemerovo databases prior to and since commencement of assistance (era A – 2000–2007; era B - 2008-2012). Totaly 1772 pediatric surgical cases were analysed. We evaluated differences between era A and era B for: Case volume per year, (+/-SD), 30 day/hospital mortality, case complexity (RACHS-1 model), and RACHS adjusted standardised mortality ratio (SMR: observed/expected mortality). For era B we evaluated year by year the number of collaborative operations where a local surgeon was primary operator.

## Results

In Era A 426 Surgeries were performed, mean annual case volume was 85 (+/- 22,8) with overall mortality of 4,9% and an SMR of 2,3. RACHS 1&2 category comprised 99,8% of the total. In Era B 1346 Surgeries were performed. Mean annual case volume increased to 269 (+/- 81,4), [p < 0.001] - with higher case complexity, an overall mortality of 3,6% and a SMR of 0,8.

In Era B 415 Surgeries were performed during 35 trips, 931 between trips. Proportion of localy lead surgeries during trips increased from 5% in 2008 to more than 60% in last 3 years of collaboration.

## Conclusion

An assistance partnership in the model applied, significantly reduced mortality, increased case volume, and complexity and developed independent operating skills in an economically disadvantaged center in a relatively short time period. This model of assistance to developing countries is not “surgical tourism”, and should always be open to scrutiny and evaluation by proven clinical and educational outcomes as presented herein.

